# Social media health promotion in South Africa: Opportunities and challenges

**DOI:** 10.4102/phcfm.v12i1.2389

**Published:** 2020-07-09

**Authors:** Brenda Z. Kubheka, Vanessa Carter, Job Mwaura

**Affiliations:** 1 Health IQ Consulting, Johannesburg, South Africa; 2 School of Public Health, Faculty of Health Sciences, University of Witwatersrand, Johannesburg, South Africa; 3 Health Care Social Media South Africa, Johannesburg, South Africa; 4 Department of Media Studies, School of Literature, Language and Media, Faculty of Humanities, University of the Witwatersrand, Johannesburg, South Africa

**Keywords:** health promotion, social media, equity, justice, digital divide

## Abstract

**Background:**

Health promotion is an effective tool for public health. It goes beyond preventing the spread of diseases and reducing the disease burden. It includes interventions encompassing the creation of supportive environments, building public health policy, developing personal skills, reorienting health services and strengthening multisectoral community actions.

**Aim:**

The aim of the review was conduct an analysis on the opportunities and challenges of the use of social media for health promotion in South Africa.

**Methods:**

A search of review articles on health promotion using social media conducted using Medline and Google Scholar. Secondary searches were conducted using references and citations from selected articles.

**Results:**

Social media has potential of being an effective health promotion tool in South Africa. It presents an opportunity for scaling health promotion programs because of its low cost, its ability to have virtual communities and the ease of access eliminating geographical barriers. It also allows real-time communication between various stakeholders. It allows information to spread far and fast and leaving irrespective of the credibility of the source of information. There is a need to take into account country specific socio-economic issues, which may perpetuate unintended consequences related to the digital divide, data costs and the varying levels of health literacy.

**Conclusion:**

Considering the opportunities presented by social media, the National Department of Health needs to review its health promotion strategy and include the use of social media as an enabler. They also need to address to explore intersectoral measures to address issues which threatening equitable access to credible health promotion information.

## Background

Innovative technologies in the media industry necessitated by the internet have offered newer perspectives in many spheres of life. Social media platforms, which have become part of the everyday lives of many individuals around the world, has provided opportunities and challenges in many areas, including healthcare. Social media platforms that include Facebook, YouTube, TikTok, Instagram, Pinterest, Twitter and even WhatsApp have become extremely popular not just in South Africa but globally and are powerful mediums of reaching billions of people. This paper reflects on the potential benefits of using social media as a tool for health promotion in South Africa, specifically looking at equitable access to credible health information and improved citizen participation within the local context.

The World Health Organization (WHO) defines health as a state of complete physical, mental and social well-being and not just the absence of disease. According to the Ottawa Charter, health promotion (HP) is the process of empowering people to increase control over and improve their health. It incorporates various disciplines, such as medicine, public health, epidemiology, psychology, sociology, social work and economics,^1,2^ and ensures that the population reaches a state of complete physical, mental and social well-being by directing interventions on an individual level.^3^ Evidently, HP’s scope goes beyond preventing the spread of disease and reducing the disease burden as it includes interventions encompassing the creation of supportive environments, building public health policy, developing personal skills, reorienting health services and strengthening multisectoral community action.^4,5,6,7^ The population should be enabled to identify their health aspirations and equipped to cope with their environment.^8^

Health is a product of a social environment, hence the call for a holistic approach to HP which ought to include mental, physical, cultural, ecological, spiritual and social aspects of health, to name a few. Effective HP strategies should be holistic and aimed at addressing context-specific determinants of health in a manner considering the local culture and belief systems. Clearly, HP strategies require multisectoral support and public commitment to facilitate successful implementation.^6^ Health promotion interventions ought to adopt a long-term perspective in building capacity to reduce the impact of social determinants of health and sustain the positive results over a long period. These interventions should lead to environmental and behavioural adaptations directed at improving health and quality of life.^2^

Both national and provincial health departments in South Africa have a considerable social media (SM) presence but with minimal HP content. There is empirical evidence proving SM interventions to be effective in inetreventions involving disadvantaged populations.^9^ The main executives in 26 out of 34 Organization for Economic Co-operation and Development (OECD) member countries maintain a Twitter account and 21 out of 36 countries maintain an official Facebook account.^10^ Social media has the capacity to reach more people faster and therefore can play a significant role as a tool for HP.^11^ The majority of studies referenced in this paper were conducted in high-income countries and therefore discount the impact of factors unique to developing countries like South Africa. These challenges include, but are not limited to, inequality, the digital divide, language barriers, gender gaps, privacy concerns, age, cultural beliefs, disability and other factors.^12,13,14^

Vitally, governments can also use SM as a new source of information allowing them to understand the needs and behaviours of people. This can be achieved through data-mining techniques such as social listening using SM analytics platforms for healthcare.^10,15^

### Ethical consideration

This article followed all ethical standards for a research without direct contact with human or animal subjects.

## Discussion

Of interest, the minister of health in South Africa had more than 90 000 followers as confirmed on Twitter on 20th March 2020, whereas the Department of Health had more than 100 000 on the same date. This shows a 33% and 44% respeviley when compared to the number of followers recorded on the 14th of July 2019. The surge in these numbers could have been as a result of the COVID-19 (Coronavirus) global pandemic, where these two social media accounts were important information resources. These numbers also indicate that on SM platforms, personalities can be as popular as the institutions they represent and also indicate that SM platforms are useful socio-political tools. In digital marketing realms, the popularity of personalities is referred to as influencer marketing and can also extend to medical professionals, patient advocates, non-profit organisations and others.^16^

Ministries, government departments and health programmes in OECD countries have a presence on SM. It is acknowledged that SM can facilitate improved communication between governments and their constituencies, enable operational capabilities and improve the governments’ responsiveness to issues raised by the public.^10^ A systemic review conducted by Moorhead et al outlined six ways in which SM can benefit public health, namely its use in surveillance, providing easy access to health information both general and tailored, interacting with various population groups, impacting public health policy and increasing social support.^17^ This echoes the potential benefits of using SM as a tool for HP.

**FIGURE 1:**
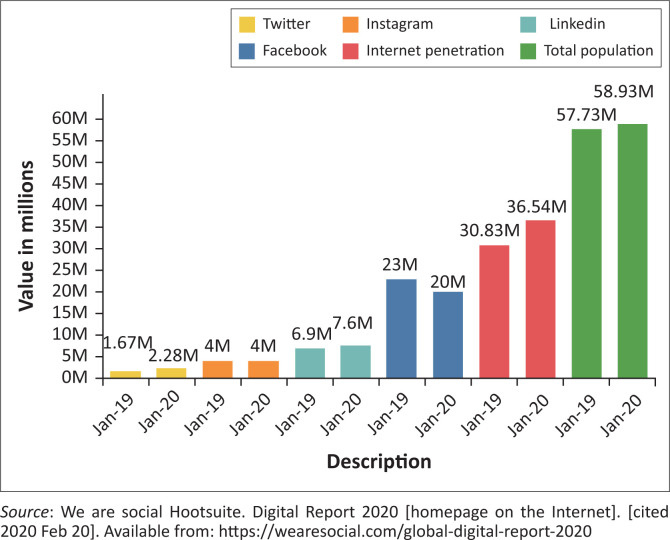
South Africa’s social media and data usage between January 2019 to January 2020.

According to a 2019 report by the Independent Communications Authority of South Africa, over 12 million South Africans use 4G/LTE devices.^18^ In another report on internet statistics in South Africa by *We are Social,* the number of internet users between January 2019 and January 2020 increased from 30.8 million to 36.5 million.^19^ The number of SM users has also been growing as shown in Figure 1.

Considering these numbers, SM presents an opportunity for HP if used constructively and in a manner that embraces the uniqueness and diversity of the South African population.

In a further reflection of these numbers on the state of the internet in South Africa, we realize how powerful SM tools can be. Social media has changed our news ecosystem more dramatically in the past few years than any other time in history. Social media has not just swallowed journalism but has swallowed everything.^19^ Social media has emerged as a powerful source of news consumption for many people.^20^ Platforms such as Twitter has not only become a place where we get breaking news, but also a space to engage in contentious socio-political engagement in real-time. During the Arab Spring in 2010 and 2011 in the Arab League, SM tools were considered as a great resource for protesters to mobilize and share information.^21^ This led to the resignation of dictatorial leaders in Tunisia and Egypt. This relates to resource mobilization theory where SM is seen as an important resource for and collective action in contemporary social movements.

It is also important to note that over the years, the media has been a powerful tool for propagating ideologies of the political class and the elite in the society, while also side-lining other groups in society. Social media has however contributed immensely in dismantling this power structures and distributing it almost evenly. There are important points which we can learn from the power of SM. Firstly, SM are not elitist driven and provide more possibilities for new forms of public participation. Secondly, SM messages can be shared with receivers/audience without the aid of intermediaries such as editors or media owners. Thirdly, there is no time delay in message sharing. Fourthly, SM offers possibilities for a multitude of voices through collective composition and meaning making (decoding) of messages. Finally, SM provides opportunities for different directions of information flow, in that a message can be shared across different SM platforms. Therefore, social media provide many opportunities for health communicators in designing and distributing messages on HP.

## Accessibility

In the United States, 79% of adults use SM and 34% of adults reported using it to obtain health information.^22,23^ A systematic and meta-analysis study reported online programmes having a high participant retention rate compared with in-person programmes.^24^ A survey conducted by Deloitte in 2010 revealed that patients with chronic illness were more likely to participate in online wellness programmes than face-to-face programmes.^23^ This may be attributed to ease of access, convenience and the minimal costs associated with their online utilisation. However, in a more dynamic and dark digital world of bots, trolls, cyborgs and deepfakes, if users are not guided on the principles of digital citizenship in terms of health information seeking, which include how to evaluate and access credible information, they are likely to become victims of fake news or alternatively reshare it.^25,26^ Therefore, one thing which should be considered during digital health transformation in South Africa are skills development programmes in public health that include citizens.

In a study by Van Rooyen on the communication of science news on SM in South Africa, the author argues that during the outbreak of Ebola SM it was shown that SM played an extremely important role in the dissemination of science-related news and information, especially if users believed it could impact their lives. […] It also served to demonstrate that Twitter had enormous potential for good science communication through the viral tweeting and retweeting. Similarly, Van Rooyen found ‘seemingly equal amounts of media coverage were devoted to the positive role […] that social media were playing in aiding the fight against the pandemic and the negative role […] that social media were playing by allowing for the rapid, rampant spread of disinformation about the disease’.^27^ This highlights the need for healthcare students, scientists and science institutions to have SM presence and, this is not an elective but a moral issue. Individuals ought to be formally trained on how to effectively use online platforms to share credible scientific information and counter disinformation, which may cause harm. Social media needs to be demystified to facilitate its adoption as an effective tool, and this is expected to change the relationship between science and the society.^28,29^

In the wake of COVID-19 pandemic, various governments around the world admitted how a huge amount of misleading information was circulating on SM about coronavirus. Often, this information contains unreliable health information which is likely to hamper and interfere with the works of health authorities and professionals. President Ramaphosa, in a speech on COVID-19 on Sunday, March 15th 2020 asked South Africans to ‘… stop spreading fake and unverified news [*which was likely to*] create further apprehension and alarm’. In a reflection by Claassen on science communication in South Africa, he argues that the media plays an important role in the dissemination of pseudoscience and this damages media reputation. He further argues that SM platforms such as Twitter and Facebook are used to spread untested and unverified health messages which sometimes go viral. He goes further to argue that the dissemination of messages on pseudoscience reinforces ‘a sort of upside-down view of how the world works, leaving people vulnerable to predatory quacks’.^30^ Therefore, it is vital that governments become aware of the changes in how people access information and the risks associated with information from sources considered not credible.

Over 1.7 million mothers in South Africa are registered with MomConnect, which is used to disseminate health information and provide support to pregnant women using various platforms such as SMS, MXit and WhatsApp.^31^ This shows that critical health information can be disseminated in ‘real-time’ and thus enable the public to engage with the relevant health promoters and the health department irrespective of geographic location. During the 2008–2009 salmonella outbreak in the United States, the Centers for Disease Control and Prevention (CDC) developed a widget that could be shared online and gave access to a database of products that had been contaminated. Members of the public were empowered to protect themselves and prevent the spread of salmonella infection.^32^ In South Africa, a significant example of this is the 2017–2018 listeriosis outbreak, which the WHO described as the ‘largest outbreak of listeriosis that has been detected globally’. It later released a toolkit in several dominant South African languages as a guide for online users to share from any location.^33^ Various health departments, the CDC, health professionals and public health activists used SM to spread information about the outbreak, affected food products and prevention measures. This resulted in SM users sharing the information and generating their own content, which included humorous memes that seemed to generate more conversations and sharing, as witnessed by the authors.

Another successful SM strategy was deployed in 2012 in the United States by the Red Cross, which utilised Twitter after Hurricane Arthur to locate critical needs without going to the sites.^34^ Evidently, SM provides individuals with access to preventative medicine information without necessarily consulting a health professional face-to-face.^35^ In terms of improving disability access to health information, SM has allowed people with disabilities to access information and it has also given them a voice they previously did not have. This is evidenced, for example, by specialised groups and communities for the deaf on platforms such as Facebook, and blind individuals benefiting from audio-visual media channels like YouTube.^36^

In essence, SM platforms can be used for various kinds of HP messages targeting different audiences. For instance, Twitter, as a microblogging platform and a social networking tool has emerged as a powerful science communication platform that more researchers embrace and is also considered more professional than Facebook, thus becoming a preferable platform for science communication.^28,37^

## Social justice

Research shows that the lesbian, gay, bisexual, transgender and queer (LGBTQ) community faces discrimination and social homophobia. This is cited as one of the barriers preventing them from seeking professional help, information and treatment insofar as their health is concerned.^38^ Social media has the potential to facilitate anonymity and make health information and services accessible, thus increasing the participation of stigmatised groups in online health conversations. These can take place without fear of discrimination and without personal contact with the provider of that health information.^39^ Moreover, SM creates accessibility to virtual community social support.

The Ottawa Health Decision Centre in Canada created a Facebook page providing shared decision-making tools to empower patients. It reported active participation in decision-making in patients who used the tools, thus increasing patients’ agency and engagement in their own care. There is evidence that engaged patients adhere to treatment better and are more informed to make treatment choices that align with their lifestyles and their desired ends.^23^ Low health literacy is considered a social determinant of health associated with poor management of chronic diseases.^40^ According to the WHO, individuals with low health literacy due to socio-economic determinants face a barrier to attaining the WHO health standard.^41^ Social media platforms and messages can be tailored in ways that help to reduce the HP disparity gap between different social and ethnic groups. Video messages can also have subtitles and thus deliver a message in multiple languages.

Communication is a barrier in healthcare generally and a serious impediment affecting access to healthcare for the deaf. The deaf community in South Africa is estimated to comprise about 1.5 million people and is underrepresented medically and socially. Its members have poorer health compared with the general population, often receive inadequate treatment or delayed diagnoses, and are even misdiagnosed. Furthermore, due to limited health knowledge, they have low adherence. Sign language in South Africa has a limited vocabulary, which impacts health communication. The United States has embarked on a successful cancer awareness campaigns through videos targeting the deaf population. The number of cell phones in South Africa exceeds the population size and they present an opportunity to boost health information campaigns, behaviour change and adherence support.^42^ Lack of health information and campaigns tailored for the deaf community is a form of discrimination termed as audism and it is unjust.^43^ Deafness and other disabilities warrant a fair chance for such persons to access health information and support to overcome their limitations.^44^

## Engagement and health literacy

SM enables individuals to create their own identities, express their opinions and participate in discussions with others, sometimes anonymously. Individuals feel they are part of a community when they are able to express themselves, be accepted, give help and get help – in other words, participate – without feeling disempowered. Public engagement is imperative for effective public health interventions.^45^ In South Africa, teenage pregnancy was recorded at 35% in 2017. A retrospective study conducted among young men between the ages of 12 and 22 who had impregnated teenage girls reported that 93.2% of the participants had more than two sexual partners, 22.4% rarely used protection and 11.5% never used protection. The study recommended that tailored sexual health and safe-sex awareness interventions need to be established for young men.^46,47^ Social media provides the opportunity to interact and engage with the target audience, who can also engage with health promoters anonymously.

According to *We are Social* January 2020 report, South Africans spend an average of 3 h and 10 min on SM a day and 9 h 22 min on the Internet a day. This is above the global average time of 2 h 24 min on social media and 6 h 43 min on the internet per day.^19^ This presents an opportunity for the government to effectively use SM as a tool for HP – with good prospects of increased public participation – and also as its source of information about the population’s health and information needs. This calls for acknowledgement of the role of SM as an enabler of HP thus requiring professionalisation of its management by individuals who possess digital marketing skills working closely with health experts or improve digital skills of health experts.

Empowerment is one of the fundamental HP principles. This requires helping the community to identify their health issues and equipping them to achieve their health aspirations. The health system has various players, and empowered patients play a critical role when they make better-informed choices.^37,49^ SM has created a platform for patients to engage with the health system. It has been reported that empowerment increases patient satisfaction. Individuals who understand health requirements are more likely to fulfil them efficiently. Healthcare information can be disseminated to individuals using SM. Individuals will not feel like patients but like team players working towards one goal along with health professionals and promoters.^49^ Social media can play a significant role in empowering patients and giving them access to reliable health information.

SM campaigns may be harder to implement than standard interventions due to a lack of strategic intent, inadequate allocation of resources and the need for expertise that is not normally found in healthcare organisations.^50^ Social media has the potential to improve access to information, enable personalised communication and increase health literacy in South Africa. It can reduce health inequalities affecting marginalised population groups like people with disabilities and LGBTQ communities.^38,51,52^

## Considerations for South Africa

### Digital divide

In a 2017 UNICEF report titled ‘mHealth and Young People in South Africa’ the cost of data and airtime was identified as the major hindrance to subscribing to online programmes. Monthly costs for data and airtime ranged from R30 to R300 and most adolescents surveyed got money from their parents, family, friends and other sources. Therefore, most young people rely on free and zero-rated services. Some of the adolescents reported that they purposefully spent time in public free Wi-Fi zones. Importantly, 84% of them reported that they would use free internet for sexual health information.^53^

The digital divide is the gap that exists between those who have access to information and communication technologies (ICT) and those who do not due to socio-economic determinants.^54^ Internet connectivity in South Africa increased from 23.9% in 2009 to 62.2% in 2017. During the same year, 70.5% of urban households had access to an internet connection versus 42.9% of rural households.^55^ Households earning more than R30 000 a month recorded 82.3% internet penetration, which is on par with developed countries and constitutes 21.5% of the South African population.^56^ Evidently, as the income per household declines, internet penetration declines as well. Limpopo and the Eastern Cape, two of the poorest provinces in the country, had the lowest proportion of households with access to the internet.^57^ High data costs are also a barrier to fully realising SM’s potential as an important tool for health education. Recently, mobile network operators reached an agreement with the Competition Commission to drop the price of data by 30% – 50%.^58^ This is a significant move because it will reduce the digital divide and widen the internet reach, which is critical for SM health promotion. The commission’s view was to elevate affordability as one of the key areas to be prioritises because access is almost universal in South Africa and the high cost being a barrier to access the internet.^59^

In contrast, poor internet and telephone network coverage in rural areas threatens the full realization of using SM tools for HP because 66% of the population in South Africa live in urban centres while 34% live in rural areas perpetuating inequality.^54^ This places a moral obligation on the government to ensure equitable access to network coverage in rural areas and this matter qualifies as a constitutional matter and a socioeconomic issue needing attention. South Africa is ranked as the most unequal country in the world. Additionally, internet access increases access to opportunities in education, knowledge and jobs, to name a few, all of which are social determinants of health. According to the World Economic Forum, an increase of 10% internet penetration contributes 2.8% to GPD growth.^57^ Therefore, internet penetration can be considered a socio-economic, political and a public health instrument.

### Language diversity in South Africa

South Africa has 11 official languages. The three most common home languages are IsiZulu, IsiXhosa and Afrikaans, in that order.^48^ English is considered the official business language and it is not surprising that majority of health information is in English. Accommodating all the official languages is a human right and an ethical issue, and it should be grounded by principles of inclusivity and non-discrimination. Social media presents an opportunity to break down language barriers because of its lower operational costs and the opportunity presented by video content, which can cater to more than one language through the use of subtitles. An opportunity exists for innovative solutions utilising artificial intelligence that enable users to choose their language and receive real-time translations.^60^

### Health promotion and social media strategy in South Africa

The 2015–2019 mHealth strategy from the national Department of Health does acknowledge the role SM platforms can play as a tool for public health promotion and education. Unfortunately, SM is omitted from the 2019–2024 national digital health strategy for South Africa.^61,62^ Social media seems to be an underutilised HP tool because its value is underestimated. To influence future policies, further research is needed to explore SM as a tool for HP, especially research based on empirical evidence. Such research ought to examine online behaviour of e-patients’ in South Africa and this can assist to determine their diverse needs for HP on SM.

## Conclusion

SM presents a significant opportunity to enhance HP programmes and campaigns and, support public health initiatives. Risks related to unvetted health information, the digital divide and health literacy issues need to be acknowledged and strategies developed to address these. Professionalising SM HP will elevate its value as a public health tool enabling real-time access to credible information. Health promotion facilitates multisectoral communication with a feedback loop on issues relevant to health and its determinants. South Africa’s HP, mHealth and national digital health strategies ought to incorporate the use of SM. This will ensure that resources get allocated to enhance online HP programmes and monitoring of their impact. The Department of Health and other stakeholders such as researchers can also extract valuable data from SM about the health and information needs of the population through analytics platforms. It can also be used to correct misinformation such as those related to anti-vaccine campaigns and COVID-19 that is spread through fake news. South Africans are already spending a significant amount of time on SM, which presents a significant opportunity for using SM platforms as a HP instrument.
